# The impact on air quality of energy saving measures in the major cities signatories of the Covenant of Mayors initiative

**DOI:** 10.1016/j.envint.2018.06.001

**Published:** 2018-09

**Authors:** Fabio Monforti-Ferrario, Albana Kona, Emanuela Peduzzi, Denise Pernigotti, Enrico Pisoni

**Affiliations:** European Commission, Joint Research Centre, Via E. Fermi 2749, TP 450, I-21027 Ispra, VA, Italy

**Keywords:** Covenant of Mayors, Air quality, Urban air pollution, Energy saving, Health impact

## Abstract

This study is a first attempt to evaluate how the major efforts made by several European cities in the frame of the Covenant of Mayors (CoM) initiative can impact the air pollution levels in the participating cities. CoM is by no mean one of the major cities initiatives aimed at mitigating climate change, supporting local authorities in the implementation of their climate action plans. Energy savings measures reported in the CoM cities' action plans have been analysed from the air quality perspective in order to find quantitative relations in the way local authorities deal with mitigation and how these practices are expected to have consequences on the air quality at urban level and finally positively impacting the citizens' health.

In the paper, the air quality 2713 energy saving measures proposed by 146 cities located in 23 countries in the frame of the CoM are selected and their co-benefits for air quality and public health estimated by means of SHERPA, a fast modelling tool that mimics the behaviour of a full physically-based Chemical Transport Model. Besides evaluating the overall benefits of this subset of mitigation measures for the air quality, the study also investigates the relevance of some factors such as the implementation sector, the city size and the pollution levels in achieving the highest possible co-benefits. The results presented refer to the special field covered by the study, i.e. energy saving measures and are not automatically referable to other types of measures. Nevertheless, they clearly show how climate mitigation and air quality policies are deeply interconnected at the urban level.

## Highlights

• Air quality co-benefits of energy saving measures in CoM are estimated.
• The analysis involves 146 cities located in 23 European countries.
• Air quality co-benefits are estimated by means of the SHERPA model.
• Co-benefits depend on the sector to which measures are applied.
• Co-benefits lead to an important amount of years of life saved.

## 1 Introduction

### 1.1 The Covenant of Mayors initiative and its tools

Recognizing the key role of cities and towns in the fight against climate change, and following the adoption of the 2020 EU Climate and Energy Package in 2008, the European Commission (EC) launched the Covenant of Mayors (CoM) initiative, to encourage local authorities to implement sustainable energy policies within their territories. CoM signatories, voluntary adhering to the initiative, commit at the moment of the adhesion to the initiative to reduce the levels of CO_2_ emissions in their territories by at least 20% in 2020 or by at least 40% by 2030, through the implementation of a climate action plan, called Sustainable Energy Action Plan (SEAP). Recently, in addition to actions on mitigation also action on adaptation (climate risk assessment) have been included, leading to the so called Sustainable Energy and Climate Action Plan (SECAP) (Bertoldi et al., 2018). In this contribution we focus on climate mitigation action plans with commitment targets for 2020, i.e. the SEAPs.

The CoM framework foresees a three steps approach: carrying out an emission inventory, setting mitigation target as well as drawing a climate action plan and lastly, monitoring the progress towards the targets. The philosophy underpinning the CoM is that, based on the emission related to final energy consumption, local authorities are able to tailor the necessary actions for implementing energy savings and increasing the renewable energy deployment in their territories (Bertoldi et al., 2010).

The inventory for accounting the emissions, named Baseline Emission Inventory (BEI) sets the principles and the minimum requirements on: the sources (activity data and related emissions in the building and transport sectors); the type of gases (only CO_2_ reporting is mandatory, but also they can report emissions of methane (CH_4_) and nitrous oxide (N_2_O)) and the boundary of the inventory.

Regarding the action plan, the SEAP comprehends the overall strategy for mitigating climate change by 2020, which is translated into a set of planned actions reported in the CoM platform. For each planned action, the signatories report the area of intervention (e.g. energy efficiency in buildings, equipment and facilities, transportation, renewable deployment, urban planning, etc.), indicating the policy instrument (distinguishing between the national/regional and the local ones) the responsible body (specific department of the local authority or other private or public entity responsible for the action) and the quantitative indicators. The qualitative indicators per each action refer to: the implementation costs, the planned energy savings and renewable production, and the estimated CO_2_ emissions reduction by 2020. At the moment of writing, 5491 SEAPs were already submitted mainly from European cities (Kona et al., 2016).

In this contribution, we will analyse a sample of 173 CoM signatories with >50,000 inhabitants, with a submitted emission inventory (i.e. BEI) and an action plan (i.e. SEAP) as of September 2016 in the CoM framework.

### 1.2 Study rationale and novelty

Recently, efforts have been made at international and European level to implement air quality and climate policies in an integrated manner (Amann et al., 2011), although such an integrated approach is still far from being implemented at local/urban scale (Viaene et al., 2016). This paper represents a first attempt to contribute to this debate by addressing for the first time the issue of co-benefits and trade-offs of the Covenant of Mayors initiative on local air quality.

Strictly speaking, some CoM signatories have already discussed synergies and trade-offs between Climate Change (CC) mitigation and Air Quality (AQ) policies in their SEAP (e.g., Barcelona in Spain, Ghent in Belgium, Bristol in United Kingdom and others). However, this study takes a more general approach, as synergies and trade-offs are looked for not in a single city, but in a large set of cities among the major CoM signatories. The overall goal of the study is to find quantitative relations in the way local authorities deal with CC mitigation and how these practices are expected to have consequences on the AQ at urban level and finally on the citizens health. For the first time to our knowledge, the mitigation measures proposed in the SEAPs of the selected signatories have been evaluated from the AQ perspective and their co-benefits for urban air pollution have been quantified, in terms of both reduced key pollutants concentrations and positive impact on public health.

More in detail, the study develops an “air quality co-benefits” indicator for a precise subset of measures detailed in the SEAPs of major CoM signatories and explores the coherence of the CC mitigation measures with the possible AQ benefits and the AQ situation of the signatories. In particular, the study focuses on the measures that mitigate CC through a decreased energy consumption pattern. For these measures, the correlation between the amount of energy saved and the AQ benefits will be assessed and a linear model will be proposed.

The relatively large number of CoM signatories included in the study, selected from the major ones, assures a robust basis to the statistical analysis performed.

The results of the study are analysed from the point of view of cities willing to develop emission reduction plans. The conclusions drawn from this study can be of support to local administrators willing to deepen and better exploit the interplay between air pollution control measures and mitigation measures in their area of responsibility.

### 1.3 Structure of the paper

Section 2 introduces the methodology of the study: available data and their sources are presented and a subset of CC mitigation measures, suitable for the analysis is defined. Finally, indicators are defined to investigate synergies between CC, AQ and public health consequences of the selected SEAPs measures.

Section 3 provides a general overview of SEAP mitigation measures together with a few statistical elaborations on the related GreenHouse Gas (GHG) avoided emissions, to be used as a basis for further investigations carried out in the following sections.

Section 4 is the core of the study. AQ and CC benefits are investigated at the sectorial level by means of the indicators introduced in Section 2. The analysis also clarifies the importance of some context variables (namely size of the cities and pollution level). Finally, the last paragraph of this section is devoted to quantifying the overall benefits of the selected mitigation measures in terms of public health gains, putting them in the context of the air pollution health impact in Europe.

Discussion and conclusions, in 5, 6, focus on the main findings and discuss the challenges of moving towards a more complete analysis in future studies and emphasise the added value of the study for city planners.

The Appendix A provides additional details on data preparation and sources.

## 2 Data preparation, indicators definition and study methodology

### 2.1 Cities selection

In order to concentrate on larger urban areas where GHG emission control policies are expected to result in a tangible impact on background air quality, a subset of the largest CoM signatory cities was selected, on the basis of the following criteria:

-the signatory has presented a full SEAP including a BEI;

-the signatory belongs to the list of cities and greater cities in Europe,^1^ Norway and Switzerland;

Following these criteria, 231 CoM signatories have been selected which also belong to the Eurostat datasets of Cities and Greater Cities. i.e. 28% of the total number of cities, representing 56% of the Cities and Greater Cities' population in 2011. For each of these cities the latest available data on population (2011) and the geographical location (lat/lon) of the cities' “centroid” was obtained from the Eurostat database. It is worth noticing that, although the cities selected are among the largest ones, the population of this sample of cities still varies considerably, ranging from about 49,000 to 6.6 million inhabitants.

### 2.2 Data preparation

A brief overview of the main data sources employed in the study and data preparation for the study follows. The Appendix A provides a detailed description of data record extracted from the listed database and more details on their merging.

#### 2.2.1 The CoM databases

The Joint Research Centre (JRC) of the European Commission has the task of checking and validating the information uploaded to the CoM platform by signatories.

The SEAP database contains a detailed description of the proposed measures and is built on information provided directly by the signatories based on their own estimates of the actual implementation and impact of the measures. Experience has shown that, due to the voluntary nature of the initiative, the difficulty of adapting local specificities to the CoM reporting framework, and the occurrence of data entry errors, not all the information collected on the Covenant platform can be considered complete and reliable. For this reason the cleaning algorithm described in (Kona et al., 2016) was preventively applied to the SEAP database before extracting the data used in this study.

The CoM related datasets also contain quantitative summaries of the Baseline Emission Inventories submitted by the CoM signatories.

#### 2.2.2 The AQ Database

This database contains values of annual average urban background concentrations of nitrogen dioxide (NO_2_) and particulate matter of diameter smaller than 2.5 μm (PM_2.5_) for most of the selected cities. This is an internal JRC database produced using SHERPA (Screening for High Emission Reduction Potential on Air), a fast tool that mimics the behaviour of a full Physically-based Chemical Transport Model (CTM). SHERPA is developed by JRC to support EC and local authorities in evaluating the impact of regional/local policies on air quality. Details on the model can be found in (Clappier et al., 2015; Pisoni et al., 2017; Thunis et al., 2016) and a recent application in Thunis et al. (2018).

#### 2.2.3 The SCE Database

This database contains the annual average Source Contribute Estimate (SCE) as % of PM_2.5_ and NOx total modelled mass. For each of the selected cities, SHERPA has been used to evaluate the percentage contributions of different sectors (e.g. transport, buildings, industry etc.) to the urban background pollution levels. SCEs are presented for the NOx, rather than directly NO_2_, because, for NOx, the contributions from the sectors can be considered linear with respect to the total concentration. Contributions are calculated considering emissions at urban background level only, i.e.., the emissions that, in principle, could be managed by local authorities. In this study, the contributions to air quality levels take into account the emissions located in the urban areas, identified according to the EU-OECD definition of Functional Urban Area (FUA),^2^ which includes the commuting zones around cities. This choice has been done to apply a harmonized definition of cities across Europe, and to make them comparable among each other.

#### 2.2.4 The HI Database

Finally, a database of Baseline parameters (e.g. mortality) for each country considered is built to allow the estimate of the Health Impact (HI) of PM_2.5_, given the Risk Rate (RR) suggested in the World health Organization (WHO) guidelines (World Health Organization, 2013).

The listed databases were merged and cleaned in order to have, for each SEAP measure, the necessary data to perform the analyses described in the following sections (see Appendix A for further details). After these procedures, a consistent dataset containing 4220 SEAP measures involving 173 cities from 24 countries was available for further analysis.

### 2.3 The challenge of quantifying the air quality co-benefits of SEAPs

The measures contained in SEAPs aim to achieve the final goal of GHG mitigation by acting on the energy profile of the signatories: by decreasing energy consumption (Energy Saving measures: shortly ES), by means of increasing the production of renewable energy at the local level (Renewable Energy Production measures: shortly REP), or both (MIX measures). SEAPs contain a translation of each measure into GHG emission reductions but no information about the expected impact on the urban AQ.

A precise estimation of the impact of a SEAP measure on the overall levels of air pollutants is impossible to be achieved on the basis of the SEAPs data only, for several reasons. First, emission factors for air pollutants, and their emission reductions, are not provided in the SEAPs as they depend both on the fuel and technology mix. Secondly, the contribution of local emissions to air pollutants concentration is difficult to estimate, as it depends on the city's actual configuration, its local and regional meteorology and, for secondary pollutants, on the chemical behaviour of precursors in the atmosphere. Finally, it is well known that air pollution in cities results from the addition of different sectoral and spatial components (transport, residential heating etc. and regional, local emissions) and again the relative weight of the components is generally different in different cities.

Nevertheless, at least at a qualitative level, it is quite clear that most of GHG mitigation measures can be expected to have a positive influence on AQ control, especially whenever energy consumption is targeted: less energy consumption in principle translates into less emissions of both greenhouse gases and air pollutants. Conversely, measures based on enhanced energy production from Renewable Energy Sources (RES), do not always translate into an AQ improvement: while wind, solar and hydropower emit very limited amounts of air pollutants, at least in comparison with the fossil fuels they typically replace in SEAPs, this is neither true for biofuels nor even more so for solid biomass burning. Emissions of some pollutants from biofuels and biomass critically depend on the combustion technology and abatement measures and are often far from being negligible or evidently lower than the fossil fuel equivalent.

**Fig. 1:**
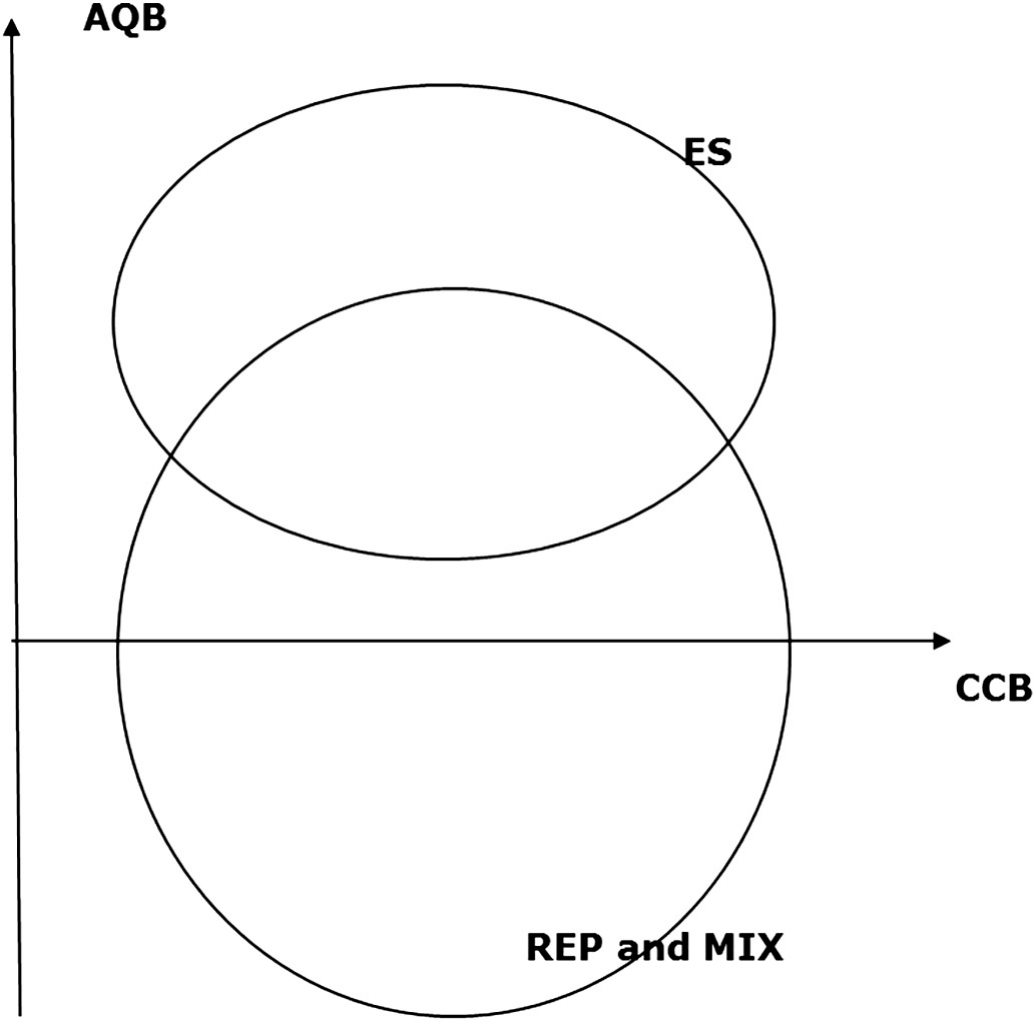
Qualitative representation of Air Quality Benefit (AQB) and Climate Change Benefit (CCB) of different kinds of SEAP measures. ES means ‘energy savings’, while REP describes ‘renewable energies production’.

Fig. 1, adapted from (von Schneidemesser and Monks, 2013), depicts qualitatively the situation: SEAP measures, that are supposed to benefit GHG mitigation (CCB > 0 - where CCB stands for Climate Change Benefit) could fall in the upper quadrant (AQB > 0 - where AQB stand for Air Quality Benefit) or lower quadrant (AQB < 0). This depends mostly on their nature: pure ES measures will certainly belong to the AQB > 0 zone, while REP and MIX measures could in principle fall in both the upper or lower area depending on the fuel used, the fuel mix substituted and, in the case of MIX measures, the relative contribution from ES and REP.

A key challenge in moving from a qualitative analysis to a more quantitative AQB estimation is that of putting a scale on the vertical axis of Fig. 1. However, while the efficacy of a SEAP measure in tackling CC can be assessed relatively easily using the known emission reductions (either absolute, fractional, per capita or in relation to its subsector emissions), for the AQ improvement potential, several of the factors previously discussed are not quantifiable using the datasets described in paragraph 2.2.

An “air quality co-benefits” indicator has been developed initially just for the ES measures, an AQB quantification of REP and MIX measures, is outside the scope of the present study, in the absence of more precise information on their nature, but it is intended to be the subject of future work. Summary data for REP and MIX measures have been in any case reported in Section 3.

### 2.4 The air quality co-benefits indicators

The “relevance” of an effective AQ measure is clearly related to its success in decreasing the average concentration of a given pollutant in a target area, in our case the selected cities. As discussed earlier, SEAPs neither provide details of the changes in emissions of air pollution precursors nor detailed information on the technologies used and corresponding fuel types for each measure.

However, in the case of ES measures, it is possible to estimate the reduction of air pollution precursors under the assumption that their reduction is proportional to the reduction in the activity level (i.e., the energy saved) in each sector. This corresponds to assuming that, for each sector, the reduction in activity does not modify the fuel/technology mix from the BEI and that a decreased activity immediately reflects in decreased emissions. Furthermore, it is assumed that the energy consumption of the sector, as reported in the BEI, is a good representation of the total energy consumed in that sector in the area of influence of the local authority. Without more detailed data, this seems a reasonable assumption for a first guess approximation to the reduction in emissions of AQ precursors. Under this assumption, it is therefore possible to define the AQ benefit of ES measures through an indicator combining, for each signatory, two aspects:

- The fraction of energy saved by the measure with respect to the energy used in the corresponding sector: the reduction in precursors' emissions and therefore the AQ benefit are considered proportional to the energy saved.

- The contribution to the AQ level (i.e., PM_2.5_ or NOx concentration) of the sector is weighted with the contribution of all sectors to AQ (as said earlier, and as an approximation this corresponds to the emissions in the area of the FUA).

To summarize these aspects the AQB (Air Quality Benefit) indicator of each ES measure applied in a city X was computed as:

(1) $$\mathrm{AQB}=\left(E_{\mathrm{SAV}}/E_{\mathrm{SEC},X}\right)*\left(C_{\mathrm{SEC},X}/C_{\mathrm{TOT},X}\right)$$

where:

• E_SAV_ = Energy saved by the measure, obtained from SEAP database
• E_SEC,X_ = Energy consumption in the city X in the sector to which the measure is applied, obtained from BEI database
• C_SEC,X_ = Contribute to the pollutant in city X of the urban emissions of the sector to which the measure is applied, obtained from the SCE database
• C_TOT,X_ = Sum of SCEs in city X of the urban emissions from all of the four sectors contained in the SCE database.

The AQB is intrinsically normalized between 0 and 1,^3^ with the maximum value corresponding to an ideal measure that saves all the energy consumed in the sole sector responsible for urban pollution in city X. In practice, ideal measures with AQB = 1 do not exist and the actual range of the resulting indicator is noticeably narrower than the normalized range. AQB can be interpreted as a measure of the impact of each SEAP measure on the fraction of pollution levels that *local authorities have the possibility to influence*.

Two AQB indicators have been associated to each measure: AQB_PM2.5_ and AQB_NOX_, referring respectively to their impact on PM_2.5_ and on NO_X_ urban background concentrations.

### 2.5 Choosing a consistent climate change benefits indicator for energy saving measures

As stated in the previous paragraph, the SEAPs database, combined with the BEI data, provides enough data to quantify the benefits of measures in mitigating climate change and some indicators such as the energy savings or the avoided CO_2_ emissions per town. In this study, the Climate Change Benefit (CCB) is simply defined for each SEAP measure by strict analogy with the AQB as:

(2) $$\mathrm{CCB}={\mathrm{EM}}_{\mathrm{SAV}}/{\mathrm{EM}}_{\mathrm{TOT},X}$$

where:

• EM_SAV_ = Equivalent emissions saved by the E_SAV_ previously described
• EM_TOT_ = Total CO_2_ Emissions in city X, obtained from BEI database

The CCB is a straightforward measure of the impact of each SEAP measure on the overall CC related emissions of the city as accounted for in the BEI. As the BEI quantifies the fraction of urban CC emissions arising from activities in the sphere of influence of the local authorities, the CCB indicator can be read as the estimate of the impact of each SEAP measure on the CC related emissions on which local authorities could have impact. From this point of view, the CCB is equivalent to the AQB, which also quantifies the impact of SEAP measures on AQ on which local authorities could in principle exercise an influence.

### 2.6 Public health impact of the decreased AQ emissions

Air quality co-benefits obtained through ES measures were finally converted into estimate on premature deaths saved and years of life gained through the standard procedure used e.g., by WHO (World Health Organization, 2013): changes in air quality levels are multiplied by risk factors and applied to a reference population.

In this way, the average number of premature deaths per inhabitant avoided due to each ES measure implemented in city X located in country C was quantified as

(3) $$\begin{array}{l} {\mathrm{PD}}_{\mathrm{AV}}=\Delta {\mathrm{PM}}_{2.5}*{\mathrm{DR}}_{C}*P_{30,C}*{\mathrm{AF}}_{2.5,\mathrm{AV}}=\left(E_{\mathrm{SAV}}/E_{\mathrm{SEC},X}\right)*C_{\mathrm{SEC},X}*{\mathrm{PM}}_{2.5,X}*{\mathrm{DR}}_{C}*P_{30,C}*\\ {\mathrm{AF}}_{2.5,\mathrm{AV}}=\mathrm{AQB}*C_{\mathrm{TOT},X}*{\mathrm{PM}}_{2.5,X}*{\mathrm{DR}}_{C}*P_{30,C}*{\mathrm{AF}}_{2.5,\mathrm{AV}} \end{array}$$

where:

• ΔPM_2.5_ is the change (decrease) in PM_2.5_ concentration attributable to the measure.
• AQB, E_SAV_, E_SEC,X_, C_SEC,X_ and C_TOT,X_ have been already defined in paragraph 2.4
• PM_2.5,X_ = average yearly urban background concentration of PM_2.5_ in city X, from AQ database
• DR_C_ = death rate in country C for all causes and above 30 years of age, from HI database
• P_30,C_ = fraction of population above 30 years of age in country C from HI database
• AF_2.5,AV_ = fraction of the mortalities attributable to air pollution calculated from the average risk factor (RF) for PM_2.5_ as (RF_X_-1)/RF_X_ where the estimated of RF_X_ is equal to 1.062^(PM_2.5,X_ / 10), with a 95% confidence interval of (1.04^(PM_2.5,X_ / 10) ÷ 1.083^(PM_2.5,X_ / 10)), as suggested by (World Health Organization, 2013).

Only avoided premature deaths related to PM_2.5_ co-benefits have been assessed because this pollutant is known to account for a number of premature deaths approximately one order of magnitude larger than for other pollutants (World Health Organization, 2013).

Finally also the avoided Years of Life Lost (i.e., Years of Life Saved) per year of exposure have been quantified for each measure through the simplified relationship:

(4) $$\mathrm{YLS}={\mathrm{PD}}_{\mathrm{AV}}*{\mathrm{YL}}_{C}$$

where PD_AV_ results from (3) and YL_C_ is the average number of life years lost per premature death in country C obtained from the HI database.

### 2.7 Investigating the link between climate, air quality and health benefits

Following the methodology defined in the previous paragraphs, it has been possible to associate to each ES measure 4 quantitative indicators, namely CCB, AQB, PD and YLS.

Analyzing the values of PD and YLS leads to the overall quantification of the health benefits gained through the CoM energy savings measures in the target cities. This result, reported in paragraph 4.4 constitutes a first key and novel outcome of this study but the quantification of health gains is not the only original finding of the study.

Thanks to the relatively high number of measures studied, it is also feasible to investigate the statistical features of the indicator sets in order to enucleate possible regularities in terms of correlations or other functional relations.

Indeed, comparing Eqs. (1), (2), (3) it is clear that both CC, AQ and health indicators are defined proportional to the energy saving, representing the actual fact that saving energy leads to GHG emission decrease, AQ improvement and public health benefits. For this reason, indicators are in principle expected to be correlated each other and, given the linear nature of the cited equations, their quantitative relations to be well described by a linear model (without intercept).

This is exactly true for measures acting on the same sector in the same city, for which all the parameters included in (1), (2), (3) assume the same value, but it is not necessarily true if measures involving different sectors and/or different cities located in different countries. In other words, the same amount of energy saved translates into a different value of CCB, AQB, PD and YLS depending on the sector, the city and the country in which such an energy saving takes place.

This leads to some questions:

- Does sector matters? Is there a sector in which CC measures translate in higher AQB than in the other sectors?

- Does city size matters? Does the same CCB leads to similar AQB, regardless the city size?

- Does the pollution level matters? Does the same CCB leads to similar AQB in both polluted and less polluted cities?

The study provides answers to these questions in paragraphs 4.1 to 4.3 applying well known statistical methodologies to the unique dataset that CoM provides.

The possible use of these results and their extension beyond the dataset considered here is discussed in Section 5.

## 3 Mitigation measures – a quantitative description

In order to provide to the reader a deeper view of the mitigation measures considered in the study, some descriptive and inferential analyses of the available data are presented in this section.

### 3.1 Mitigation measures overview

After the dataset merging and cleaning procedures, 4220 mitigation measures were selected for further analysis: the largest share (2275 – 54%) involves the Building sector, while 1054 (25%) are directed at the Traffic sector, 785 (19%) at Industry and 106 (2%) to “Other”.

Following the definition of energy savings, renewable energy production and mixed saving and renewable measures in paragraph 2.3, 2721 measures fall in the ES category, 526 are classified as REP, 205 as MIX while for 768 measures a zero or missing value for both energy saving and renewable energy production records was found, not allowing their classification in one of the previously defined categories. Among the ES measures, 8 were excluded because the declared amount of energy saved was larger than the whole consumption of the target sector, leading to a final sample of 2713 ES measures, proposed by 146 cities located in 23 countries.

It is worth also noticing that the energy saving measures are the category of mitigation measures leading to the highest amount of CO_2_ emission reduction (18.242 M tCO_2_-eq/year) while REP measures will account for a reduction of 4.892 M tCO_2_-eq/year and MIX measures for 3.01 4.892 M tCO_2_-eq/year.

**Fig. 2:**
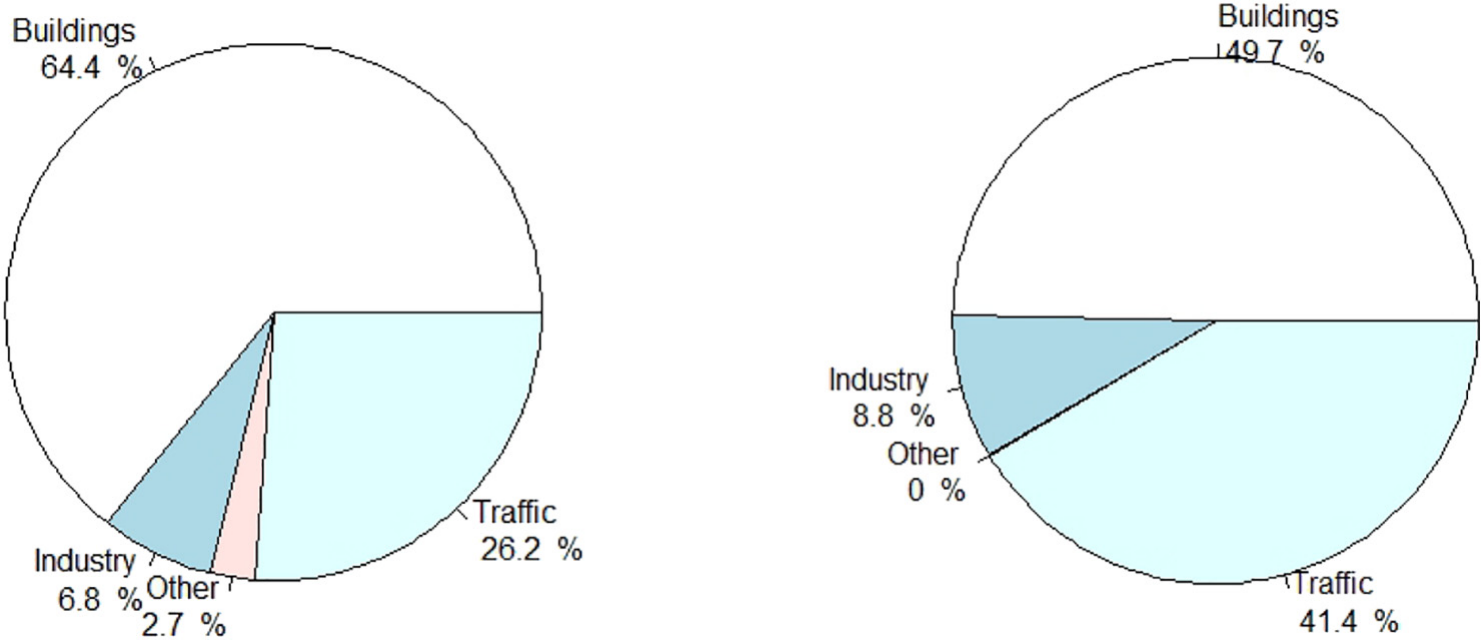
Share of ES measures (left) and energy saved (right) per sector.

Fig. 2 shows the share of ES measures (left) and the share of the overall amount of energy saved by the selected measures per sector (right): ES measures targeting buildings sectors account for almost two thirds of the selected ES measures but for only half of the overall energy saved. Conversely, traffic ES measures count for slightly more than one quarter of the measures selected yet account for >40% of the overall energy saved.

As far as countries involved, the 146 cities are unequally spread among European countries, with Spain and Italy accounting for 38 and 34 cities respectively, Germany for 11 and all other countries for <10 cities each. The selected cities have different sizes, with populations ranging from 6.6 million inhabitants down to about 50,000, with a median value of 154,000 inhabitants. For this reason the population covered by the selected sample is distributed slightly differently to the number of cities, with Spain and Italy still leading, but followed by France and Portugal, and Germany being only fifth. Further details on the number of ES measures, overall energy saved and CO_2_ emissions avoided are reported in Table 1.

**Table 1:** Per county distribution of CoM signatories cities selected for this study. Cumulative values of population, number of ES measures implemented, saved energy and CO_2_ emissions avoided are also reported.

Country	Number of signatories	Population	Number of Energy Savings measures	Total estimated energy savings by 2020 [TWh/year]	Total estimated CO_2_ emission reduction by 2020 [M tCO_2_-eq/year]
BE	4	511,164	40	1.304	0.406
BG	5	1,889,087	52	1.640	0.479
CH	1	354,755	8	0.094	0.028
CZ	1	296,324	24	0.172	0.068
DE	11	3,164,633	80	4.576	1.837
DK	1	198,401	12	0.356	0.068
EE	1	390,369	23	0.473	0.130
ES	38	13,111,263	822	12.211	4.356
FR	9	9,857,389	46	0.718	0.129
UK	7	2,312,872	78	1.787	0.458
EL	1	83,640	69	0.051	0.080
HR	3	1,022,383	93	2.084	0.616
HU	2	1,885,149	17	1.647	0.419
IT	34	11,676,675	597	22.499	5.689
LT	1	315,165	7	0.377	0.084
LV	2	136,115	36	0.147	0.033
NL	2	351,640	5	0.208	0.078
PL	3	692,953	28	0.184	0.093
PT	8	3,802,884	420	5.506	1.788
RO	8	1,191,417	205	1.347	0.616
SE	1	112,949	3	0.017	0.008
SI	1	279,624	21	1.190	0.390
SK	2	488,157	27	1.593	0.374
Total	146	54,125,008	2713	60.181	18.242

Regarding the geographical coverage, most of the CoM signatories are towns located in Southern European countries, like Italy and Spain, where dedicated bodies, including Covenant Territorial Coordinators (CTCs), have supported cities in the process of adhesion to the CoM (Kona et al., 2016).

### 3.2 Mitigation impact of energy saving measures

**Fig. 3:**
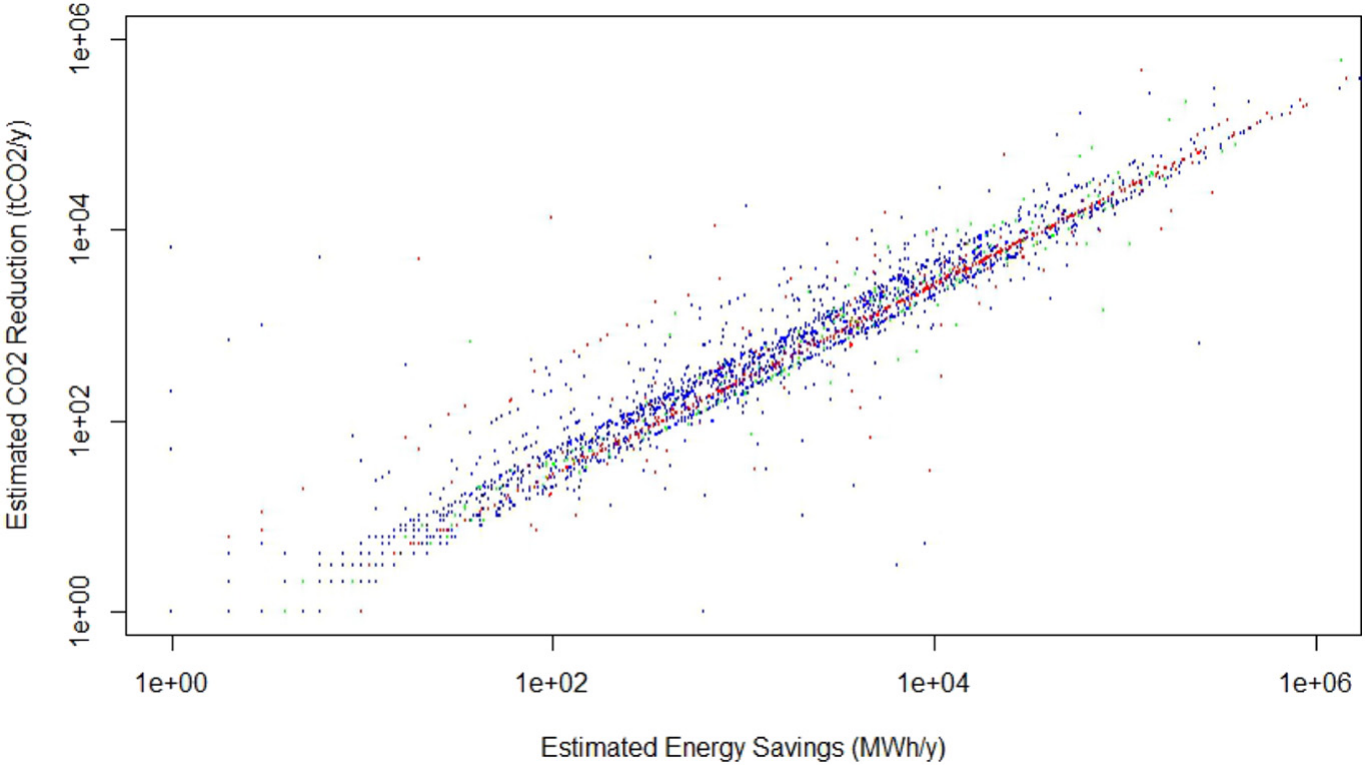
Estimated CO_2_ reduction (tCO_2_/y) versus estimated energy savings (MWh/y) for ES measures. Measures belonging to Traffic, Buildings, Industry and Other sectors are shown respectively in red, blue, green and black. (For interpretation of the references to colour in this figure legend, the reader is referred to the web version of this article.)

Fig. 3 shows the scatter plot (in logarithmic scale) of CO2 emission decrease versus energy saved for the 2713 measures discussed. Measures belonging to Traffic, Buildings, Industry and Other sectors are shown respectively in red, blue, green and black. It is worth noticing how both energy savings and estimated CO2 reductions associated with SEAP measures span more than five orders of magnitude, ranging from very limited and timely measures to wider energy consumption control strategies targeting large segments of the overall city activities.

**Fig. 4:**
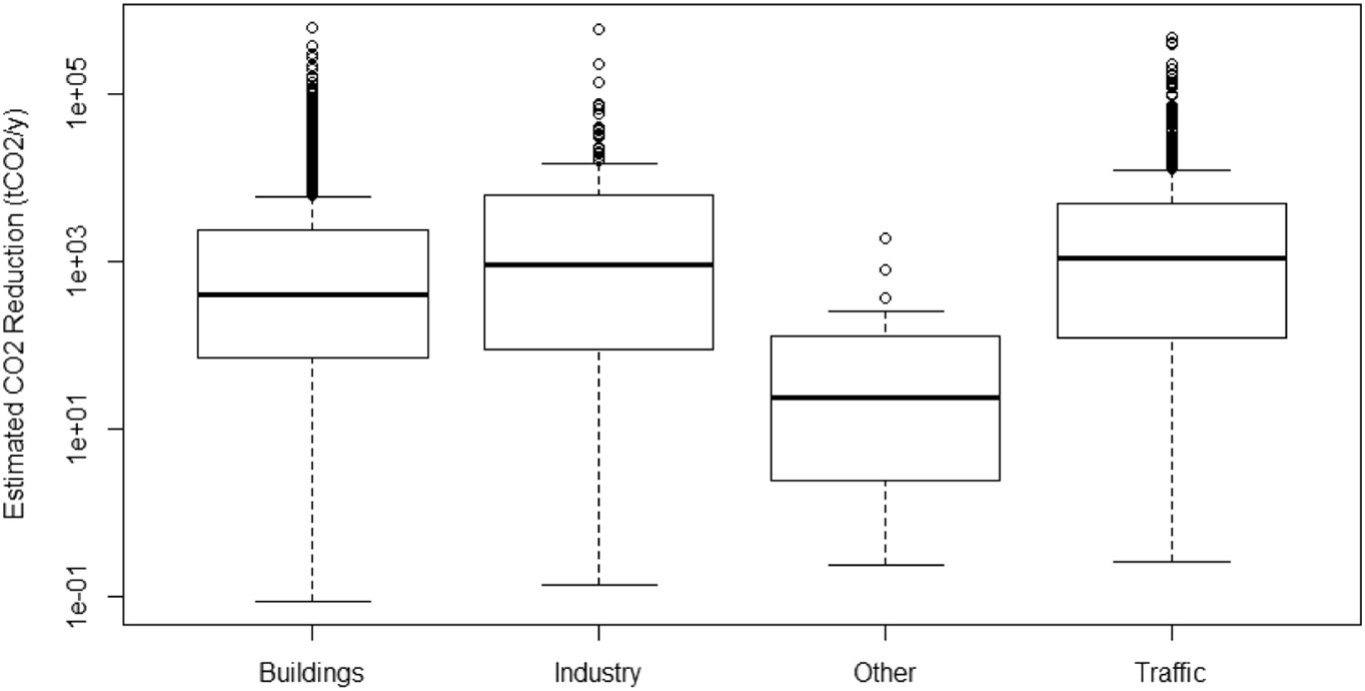
Boxplot of estimated CO_2_ reduction (tCO_2_/y) versus sector. ANOVA shows that the differences in the distributions of reductions among sectors statistically significant with p < 0.01.

A close observation of Fig. 3 shows different patterns in emission saving measures in each sector (see Fig. 4 for a boxplot) and ANOVA^4^ confirms that the sectorial distributions are statistically different with p < 0.01.

Fitting the data in Fig. 3 to a simple Y = αX model (linear with no intercept) both for the whole data set and separately for each subsector provides the values of alpha reported in Table 2 (95% level of confidence). R^2^ values for the linear fits for the whole sample as well as subsectors range from 0.75 to 0.9, providing quantitative evidence of the robustness of the linear trends clearly visible in Fig. 3.^5^

**Table 2:** Linear model fitting coefficient and R^2^ values for CO_2_ emission decrease versus energy saved.

Sector (N)	Coefficient (tCO_2_/MWh)	R^2^
Full sample (2713)	0.268 ÷ 0.278 (0,273)	0.80
Buildings (1746)	0.264 ÷ 0.275 (0.269)	0.84
Traffic (710)	0.239 ÷ 0.258 (0.248)	0.77
Industry (186)	0.390 ÷ 0.429 (0.409)	0.90

### 3.3 Effect of the city sizes on the efficacy of emission reductions

Several factors related to the city features could influence the CO_2_ reductions achieved by the SEAPs and some of them, including climatic area and degree of urbanization have been investigated in (Kona et al., 2016) and, for a slightly different subset, in (Croci et al., 2017). Here, we focus on the effect of the city size anticipating an aspect that will be discussed also in relation with AQ in the next section. In order to clarify the relation between CO_2_ reductions and the dimension of the cities, the ES measures sample has been segmented on the basis of the size of the cities in which measures were applied. Cities were divided into two groups: a first group including “larger cities” (i.e., with >200,000 inhabitants) containing 56 cities implementing 987 ES measures and a “small cities” group, (i.e., with <200,000 inhabitants) containing 90 cities applying 1726 ES measures. The distributions of absolute CO_2_ reductions and CO_2_ reductions per capita were compared between the two subsets. Quite interestingly, it was found that the two indicators behave in an opposite way: as expected the average absolute CO_2_ reduction associated to each ES measure implemented by bigger cities is larger than in smaller cities (about 44,000 tCO_2_/y versus 9000 tCO_2_/y). On the contrary the average CO_2_ reduction per capita achieved by each ES measure is smaller in larger cities and averages to 0.066 tCO_2_/(y*cap) versus the value of 0.084 tCO_2_/(y*cap) for smaller cities.^6^ Clearly, larger cities have the opportunity to plan major measures leading to important absolute CO_2_ reductions, mainly because of the larger size of activities they have under control. On the contrary, smaller cities have lower emissions that can be reduced, but seem to have the opportunity to be more effective for individual inhabitant.

## 4 Energy saving measures from the air quality perspective

### 4.1 Comparing air quality benefits and climate change benefits of ES measures

**Fig. 5:**
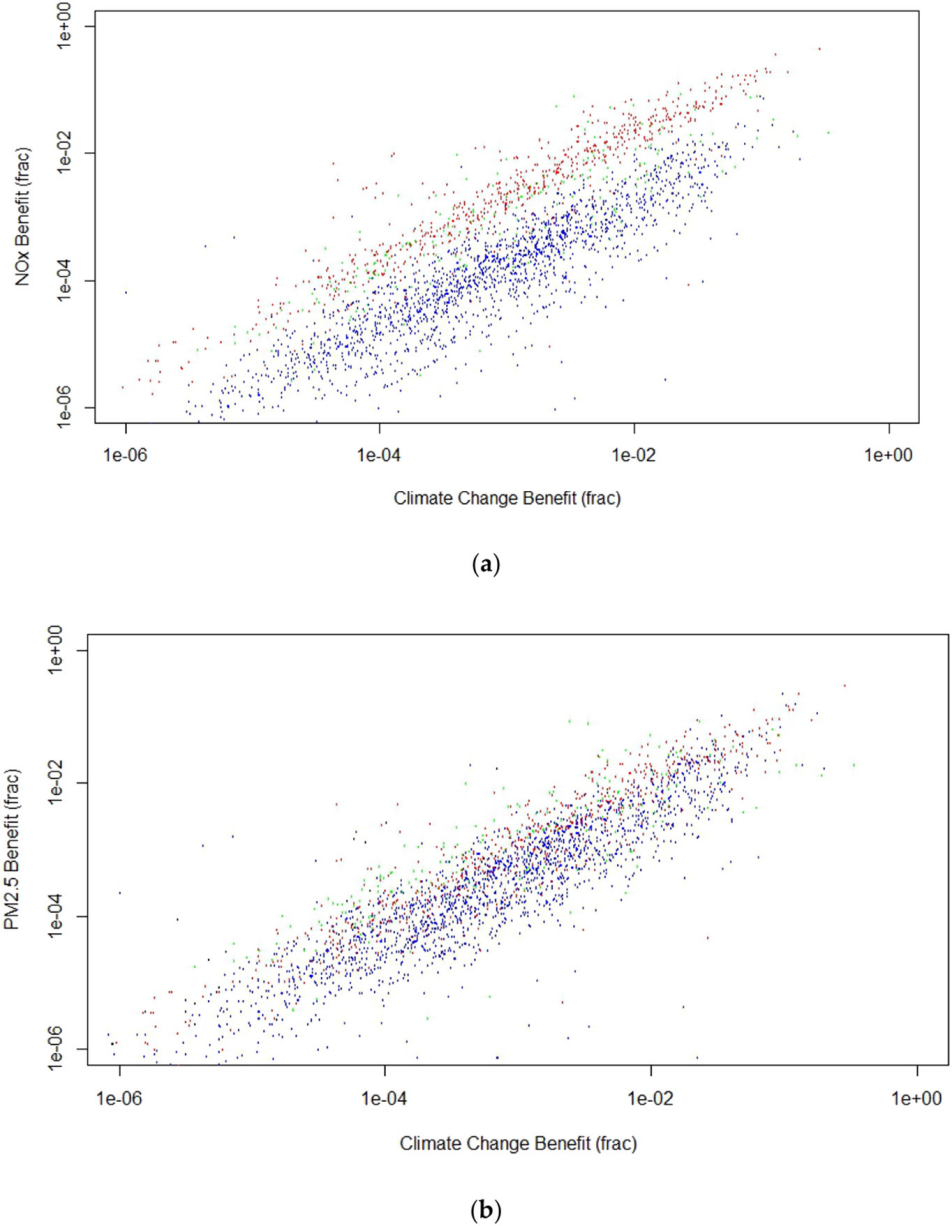
(a) AQB _NOx_; (b) AQB _PM2.5_ versus CCB for the selected SEAP measures. Colour coding as in Fig. 3. (For interpretation of the references to colour in this figure legend, the reader is referred to the web version of this article.)

Fig. 5 shows the scatter plots of AQB_NOX_ (panel a) and AQB_PM2.5_ (panel b) indicators versus CCB indicator for the 2713 measures analysed. Measures belonging to Traffic, Buildings, Industry and Other sectors are again shown respectively in red, blue, green and black. ANOVA confirmed that distributions of AQB values significantly differ among emission sectors with p < 0.01.

The simple linear model in Eq. (5) was again fitted to the whole data set and to sector subsamples.

(5) AQB=α∗CCB

Fitting results and R^2^ values are reported in Table 3 and Table 4 for NO_x_ and PM_2.5_ respectively.^7^

**Table 3:** Linear model fitting coefficient and R^2^ value for AQB_NOX_ versus CCB for the whole sample and for sectors for which a value of R^2^ > 0.4 was found.

Sector (N)	Coefficient	R^2^
Whole sample (2713)	0.743 ÷ 0.807	0.44
Buildings (1746)	0.190 ÷ 0.207	0.53
Traffic (710)	1.609 ÷ 1.707	0.86

**Table 4:** Linear model fitting coefficient and R^2^ value for AQB_PM2.5_ versus CCB for the whole sample and for sectors for which a value of R^2^ > 0.4 was found.

Sector (N)	Coefficient	R^2^
Whole sample (2713)	0.585 ÷ 0.626	0.54
Buildings (1746)	0.542 ÷ 0.591	0.54
Traffic (710)	0.879 ÷ 0.944	0.80

In practice, relation (5) implies that, on average, a SEAP measure leading to a given X% decrease of CO2 emissions in the target city is expected to also result in the decrease of the indicator of urban background AQ levels (for PM_2.5_ and NO_2_) caused by local pollutants emissions reduction of about αX%. The value of α is given in Table 3 and Table 4 as a function of the target pollutant and the sector where the CO_2_ emissions reduction takes place. It is interesting to notice that in the case of PM_2.5_ the average traffic related SEAP measure is expected to produce an AQ co-benefit per unit of climate benefit approximately 60% larger than the average buildings measures. As expected this effect is much stronger for NO_X_, with the average traffic related SEAP measure expected to produce an AQ co-benefit per unit of climate benefit 8.5 times larger than the average measures involving buildings.

It can also be noticed that the Traffic and Buildings sectors rank reversely than in Table 2, where the building sector provides a slightly larger CO_2_ emission reduction per unit of energy saved than for Traffic.

In general terms, this implies that the most effective sectors to be targeted with ES measures differ according to the target: CC control is slightly better achieved with measures targeting the buildings sector, while AQ control is much more efficiently pursued saving energy in the Transport sector at urban level.

### 4.2 AQ co-benefits vs. city size

Similarly to CO_2_ emission reductions, the influence of city size on AQ co-benefits was also investigated. Table 5 and Table 6 shows the results obtained fitting again a linear model when the same city size based sample segmentation described in the previous paragraph is applied. (Industry is again excluded because of low R^2^ values).

**Table 5:** 95% confidence intervals for AQB_PM2.5_ fitting CCB through Eq. (5) in case of ES measures segmented by both sector and city size. Subsamples size is reported in parentheses.

	>200 k inhabitants	<200 k inhabitants
All sectors	0.536 ÷ 0.598 (987)	0.593 ÷ 0.648 (1726)
Traffic	0.768 ÷ 0.857 (308)	0.891 ÷ 0.980 (402)
Buildings	0.404 ÷ 0.469 (580)	0.672 ÷ 0.741 (1166)

**Table 6:** 95% confidence intervals for AQB_NOx_ fitting CCB through Eq. (3) in case of ES measures segmented by sector and city size. Subsamples size is reported in parentheses.

	>200 k inhabitants	<200 k inhabitants
All sectors	0.565 ÷ 0.668 (987)	0.796 ÷ 0.876 (1726)
Traffic	1.548 ÷ 1.719 (308)	1.602 ÷ 1.726 (402)
Buildings	0.151 ÷ 0.181 (580)	0.223 ÷ 0.244 (1166)

Table 5 and Table 6 show how values of α are generally higher for smaller cities, for all sectors and pollutants considered. It seems that smaller cities are more efficient in converting CC benefits into AQ benefits, similarly to what has been shown in Section 3 about the better ability of small cities to work more efficiently on CO_2_ reductions in per capita terms.

### 4.3 Air quality co-benefits versus pollution levels

A further analysis concerned the relation between the amount of AQ co-benefits provided by ES SEAP measures and the state of air quality in the cities considered in the study. Cities were divided in two subsamples on the bases of the NO_x_ and PM_2.5_ concentrations reported in the AQ database: cities with “High NO_x_” levels were identified as cities showing an average urban background NO_x_ concentration above the median value, while remaining cities were denoted as “Low NO_x_” cities. A similar sample segmentation was implemented for PM_2.5_.

ANOVA analysis compared the distributions values of CCB, AQB_NOX_ and AQB_PM2.5_ indicators in less polluted versus more polluted cities without finding any statistically significant difference (p < 0.01): more polluted cities implement ES measures statistically giving the same type of CC and AQ benefits as measures by less polluted cities.

### 4.4 Health impact of energy saving measures

In total, the implementation of the 2713 ES measures included in this study is expected to translate into a most probable value of 6596 avoided premature death, with a 95% confidence interval of (4356 ÷ 8572) and 68,476 Years of Life Saved with a 95% confidence interval of (45,403 ÷ 89,358). Sector breakdown is shown in Table 7. For comparison it is worth reminding that (Holland, 2014) estimates 339,008 premature deaths caused by PM_2.5_ in the EU in 2015, corresponding to about 3,394,000 Years of Life Lost.

**Table 7:** Avoided Premature Deaths and Years of Life Saved thanks to the studied SEAP measures. Total and per sector values. 95% confidence intervals are reported in parentheses.

	Avoided premature deaths	Years of life saved
All sectors	6569 (4356 ÷ 8572)	68,476 (45,403 ÷ 89,358)
Traffic	3280 (2173 ÷ 4282)	34,192 (22,658 ÷ 44,643)
Buildings	2591 (1719 ÷ 3379)	27,003 (17,914 ÷ 35,219)
Industry	673 (447 ÷ 878)	7000 (4645 ÷ 9127)

**Fig. 6:**
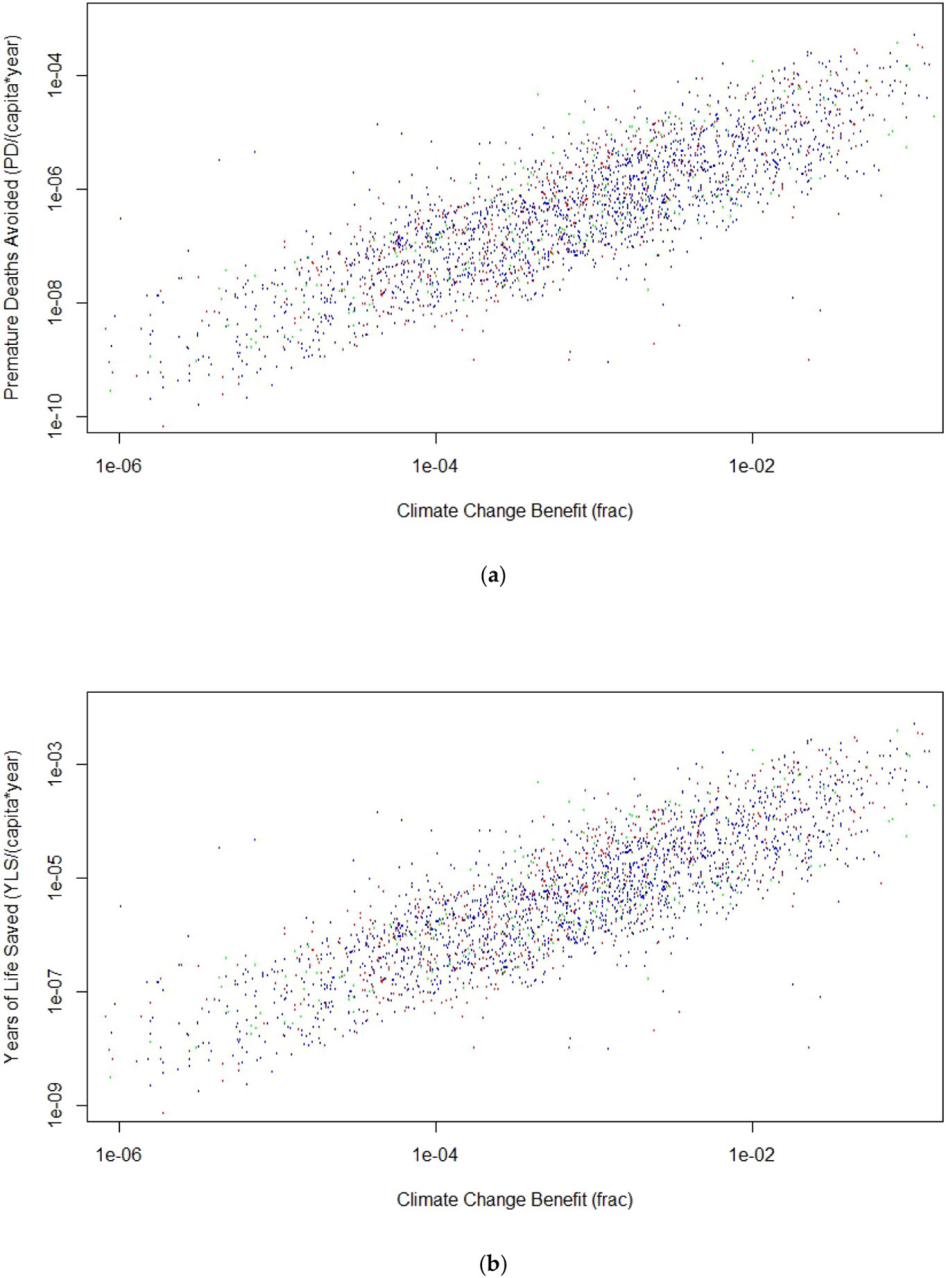
(a) Avoided premature deaths; (b) Years of Life Saved versus CCB for the selected SEAP measures. Colour coding as in Fig. 3. (For interpretation of the references to colour in this figure legend, the reader is referred to the web version of this article.)

Fig. 6 shows the premature deaths avoided per year per capita (panel a) and the year of life saved per capita per year (panel b) thanks to the reduction of PM_2.5_ levels obtained by means of the SEAP ES measures computed following Eqs. (3), (4).

For the Health Impact indicators shown Fig. 6 the ANOVA analysis did not show any statistically significant difference when the sample is segmented based on subsectors, then just the whole data samples for the PD and YLS indicators have been fitted to a linear model with no intercept finding the results reported in Table 8.

**Table 8:** 95% confidence intervals for PD and YLS fitting CCB through a linear model with no intercept. No sample segmentation was applied.

		R^2^
PD	8.27 ÷ 9.26 × 10^−4^	0.31
YLS	8.72 ÷ 9.75 × 10^−3^	0.31

## 5 Discussion

### 5.1 The AQ co-benefits of CoM initiative. A robust finding

In this work we have tried to define and quantify the AQ co-benefits achieved through the implementation of measures leading to decreased energy consumption in the frame of the Covenant of Mayors initiative. Our approach is based on the use of the SHERPA tool and relies on the basic assumption that energy savings not only translate into CO_2_ emission reductions, but also into air pollutants reductions and that emissions decrease in each sector are proportional to the actual saved energy as estimated in the SEAP presented by signatories.

In this way, each measure leading to energy savings and then to a certain climate mitigation benefit has been associated to a certain AQ co-benefit finally leading to air pollution decrease. More precisely, the co-benefits of energy saving measures on NO_X_ and PM_2.5_ on the cities pollution level have been quantified. In the case of PM_2.5_ the benefits have been also translated into impacts to public health in terms of avoided premature deaths and years of life saved. All along the analysis, a statistical approach has been taken, in order to underline the main factors determining the AQ co-benefits of SEAP measures.

A main result of this study is the demonstration of existence of co-benefits: for the cities involved, which are among the largest ones in the CoM initiative, the presence of relevant co-benefits has been demonstrated in a robust way. These co-benefits have been quantified and shown to be not negligible in comparison to the overall effect on public health of the current air pollution in Europe.

Moreover, statistical analysis has also shown that co-benefits depend on the sector targeted and the pollutant considered. In fact, given the same positive impact on climate change mitigation, at the urban level measures involving the traffic sector are expected to translate into a benefit for NO_X_ pollution about eight time higher than an equivalent measure targeting the building sector, while PM_2.5_ benefit of traffic measures is "“only"” about 60% larger than equivalent measures focusing on building sector.

AQ co-benefits have been shown to depend also on the size of the city in which the measure is implemented, while no evidence was found that energy saving measures implemented in cities affected by higher pollution result in higher air quality co-benefits.

### 5.2 Crucial points, limitations of this study and outlook

The study presented is clearly affected by many limitations and uncertainties, mainly arising from underlying uncertainties in the main input data and, to some extent, to the intrinsic uncertainties brought in by the methodology employed. The most important issues are discussed hereafter: we plan to provide better quantitative analysis of the impact of each class of uncertainties in the near future by means of appropriate sensitivity analysis.

#### 5.2.1 Data quality and statistical significance

Throughout this study, a relevant effort has been devoted to assure the quality of data analysed, especially in the case of data originating from the SEAPs database. For this reason a strict data cleaning procedure has been implemented excluding more and more records from the original dataset. Data selection on one side assures data quality and consistency while on the other side the exclusion of data comes to the expenses of significance and sample coverage. However, the size of the resulting reference sample has been judged large and diverse enough to properly represent the diversity of the actual circumstances in which SEAP measures are implemented. A geographical imbalance is evident in the CoM data, but this realistically reflects the well-known predominance of administrations from some countries in embarking themselves in the CoM initiative.

#### 5.2.2 Model robustness and emission estimates

The fundamental link between CC and AQ benefits has been obtained through the use of air quality modelling results. Robustness of the SHERPA model is discussed in other papers (Pisoni et al., 2018, Pisoni et al., 2017) but in general the percentage bias error comparing SHERPA and a full-fledged Chemical Transport Model lies around ±10%. (Pisoni et al., 2018) shows also as other major uncertainties could arise from AQ emissions estimates. The full sensitivity analysis conducted there shows that the most influential inputs on the modelled AQ concentrations are by far data contained in the emission inventory, while model coefficients are less influencing the output variability. Such uncertainties could in principle have a relevant impact on the quantitative relations between CCB and AQB. Also, SHERPA is based on Europe wide emission inventories, and not on local city level ones, that in principle are more accurate to describe local features than European ones.

Moreover, as measured data were not available for the full set of the cities analysed, the pollution levels used in paragraph 4.3 and for assessing benefits to human health in Eq. (3) have been obtained from modelling estimates. In some cases, modelling estimates are known to differ from measured values, especially in the case of PM_2.5_.

#### 5.2.3 Urban areas definition

For several cities there is a mismatch between the urban area as defined in the CoM initiative and the FUA on which SHERPA analyses are based. The CoM database, in fact, includes both cities and urban conglomerates comprised of several neighbouring municipalities who agreed to submit a single coordinated SEAP. Therefore it is not straightforward to systematically identify the areas concerned by each signatory. As said earlier, the contributions of the sectors' emissions to air quality are considered taking into account the FUA for each signatory. This choice allows more robust results (as the area of the FUA is larger than that of the city), but, on the other side, it is likely to lead to some overestimation of the AQ effect of the CoM measures. Nevertheless, this overestimation is also likely to be damped because in Eq. (1) AQ benefits are expressed as relative contributions of the sectors concerned by the ES measures.

#### 5.2.4 Time frame consistency

**Fig. 7:**
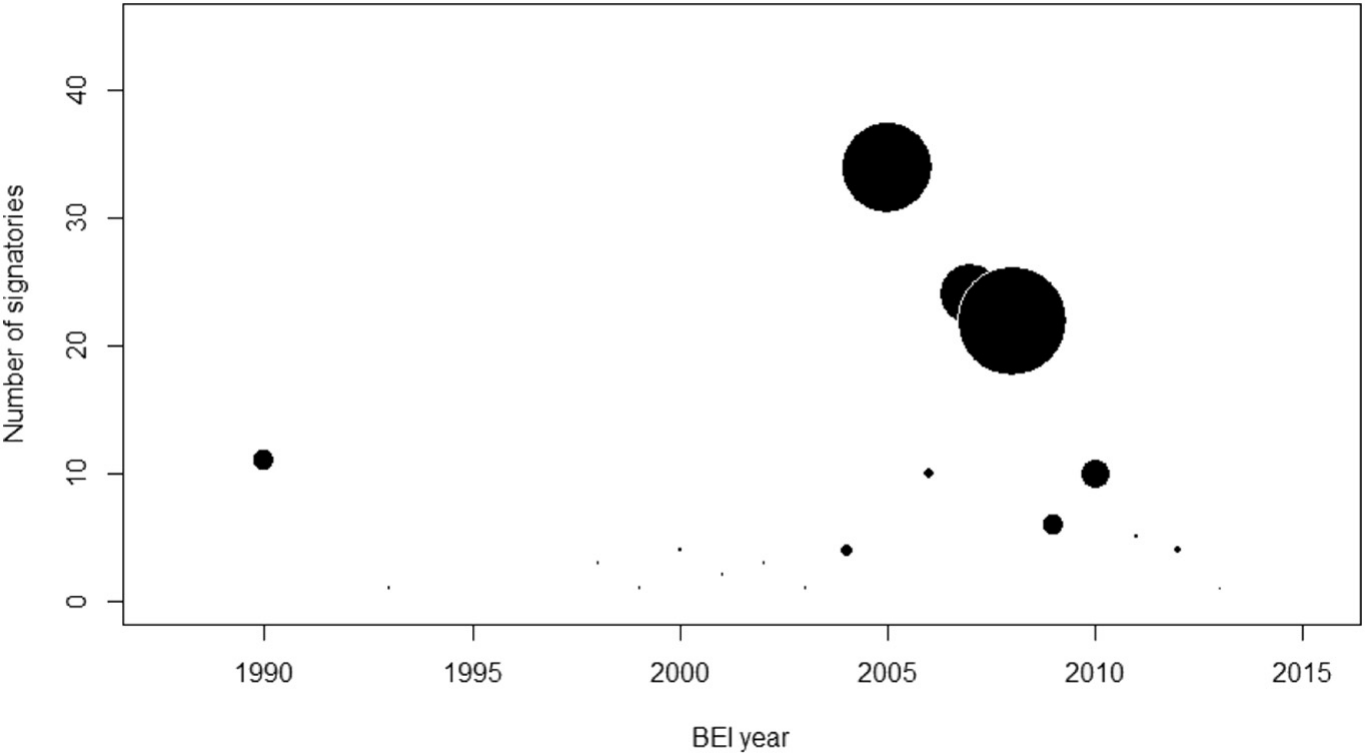
Number of selected signatories per year of BEI submission. The size of the circles is proportional to number of energy saving measures analysed in this study.

Because every CoM city selects its own baseline year, from 1990 onwards, the baseline GHG emissions of signatories selected for the sample do not necessarily match the baseline year of pollution emissions used in SHERPA for the SCE assessment, currently fixed at 2010 although time difference between the two inventories is generally limited to a few years (see Fig. 7). To provide a more robust assessment of the impact of time frame inconsistencies, a sensitivity analysis has been performed excluding signatories using a BEI reference year farer than 5 or 3 years from 2010. The key results of the present studies have shown to be substantially robust from this point of view.

#### 5.2.5 Main limitations of the study

Clearly the study suffers of the major limitation of considering only energy saving measures. as discussed paragraph 2.4, more detailed data would be required to include the whole set of CoM measures, including the ones having a possible negative effects on AQ. Nevertheless, it has to be also considered that in the frame of the CoM initiative, the largest share of CO_2_ emission reduction is obtained by means of energy saving measures.

Another major limitation consists in the strong hypothesis of considering average emissions of each precursor proportional to the energy used in each sector. Of course a more detailed definition of the sector and a deeper analysis of each and every measure would allow a more precise evaluation.

## 6 Conclusions

In this paper we have provided a first quantitative evaluation of the AQ co-benefits of energy saving measures described by the CoM, probably currently one of the largest bottom-up coordinated societal action in Europe aimed at mitigating climate change. The availability of a relatively large number of robust data on mitigation actions and the use of a state-of-the-art mature air quality model specifically designed for the urban scale provided estimates of the AQ co-benefits of policy measures originally designed with climate mitigation in mind. Statistical analysis has shown how AQ co-benefits are linearly dependent on CC benefits, with different slopes and goodness-of-fit values depending on the sector targeted and the pollutant considered.

The next natural question is to what extent the results of this study could be generalized to cities not included in the sample. The answer depends on several factors and also on what is meant by generalization. It is clearly possible to apply the methodology described and applied in this study to cities not included in the sample we have studied: the SHERPA tool is available for most of the European territory and can in principle be applied to any other city in the area. Nevertheless, in cities that are too small the influence of urban emissions on pollution levels may be too small to be clearly identified against the background and SHERPA (as any other air quality model in such a situation) is not expected to provide meaningful results.

On the contrary, one should be very careful in generalizing and extending the results provided in Fig. 5, Fig. 6 and in Table 8 on the quantitative relations between climate benefit, air quality benefits and health benefits. Moreover, one has to remember that the voluntary nature of the CoM initiative could in principle introduce an a priori bias in favour of the most environmentally virtuous cities. Finally, another level of complexity in assessing the CoM initiative results is due to the fact that some measures taken and implemented at local level by the signatories are mixed with independent regional or national measures making unclear the actual local contribution.

Nevertheless, we think the main message of our study relies in reminding the CoM signatories and the local administrators that AQ co-benefits of mitigation measures exist, could be quite relevant and, more importantly, come “for free” on top of the mitigation advantages. In paragraph 4.3 we have also shown that AQ co-benefits are generally not fully consistent with pollution levels in the cities. For this reason, we think our study could be of inspiration and guidance to CoM signatories and other administrators for better tuning and fully exploiting the AQ co-benefits of their planned mitigation measures.

Future studies should try to analyse the co-benefits of climate change mitigation measure with air quality based on the comparative analysis of CoM emission inventories and their implementation reports. A good understanding of implementation phase by analyzing in detail energy savings measures already achieved is needed to effectively replicate the results in other cities.

## List of acronyms

AQ: air quality
AQB: Air Quality Benefit
BEI: Baseline Emission Inventory
CCB: Climate Change Benefit
CoM: Covenant of Mayors
EC: European Commission
ES: Energy Saving
EU: European Union
FUA: Functional Urban Area
GHG: GreenHouse Gas
HI: Health Impact
JRC: Joint Research Centre
PD: Premature Deaths
REP: Renewable Energy Production
RR: Risk Rate
SCE: Source Contribute Estimate
SEAP: Sustainable Energy Action Plan
SECAP: Sustainable Energy and Climate Action Plan
SHERPA: Screening for High Emission Reduction Potential on Air
WHO: World Health Organization
YLS: Years of Life Saved

## Figures and Tables

**Table A.1:** Fields of the CoM SEAP database used in this study.

Field name	Description	Measure unit/categories
OID	City identifier	
URAU NAME	City name	
Subsector	CoM Activity sector on which the measure acts	See Table A.6
Estimated Energy Saving (EES)	Energy saved by the measure in the application subsector	MWh/year
Estimated Renewable Energy Production (EREP)	Renewable energy produced thanks to the measure application	MWh/year
Estimated CO_2_ Reduction	CO_2_ emissions avoided thanks to the measure application	tCO_2_-eq/year

**Table A.2:** Fields of the CoM - BEI database used in this study.

Field name	Description	Measure unit/categories
Oid	Signatory identifier	Numeric
Total emissions	Total CO_2_ emission from the signatory's emission inventory	tCO_2_-eq/year
Total final energy consumptions	Total final energy consumptions from the signatory's inventory	MWh/year
Total final energy consumptions in the subsector	Total final energy consumptions from the signatory's inventory for each subsector	MWh/year

**Table A.3:** Fields contained in the AQ database.

Field name	Description	Measure unit/categories
Oid	Signatory identifier	Numeric
NO_2_ concentration	Urban background concentration of NO_2_	μg/m^3^
PM_2.5_ concentration	Urban background concentration of PM_2.5_	μg/m^3^

**Table A.4:** Fields contained in the SCE database.

Field name	Description	Measure unit/categories
Oid	Signatory identifier	Numeric
PM_2.5_ SCE	PM_2.5_ urban background source apportionment for SHERPA sectors (see Table A.6 for the sectors list)	%
NO_x_ SCE	NO_X_ urban background source apportionment for SHERPA sectors (see Table A.6 for the sectors list)	%

**Table A.5:** Fields contained in the HI database. Source: WHO mortality and demographic tables (available at http://data.euro.who.int/dmdb).

Field name	Description	Measure unit/categories
CNTR	Country identifier	Numeric
Death rate	Fraction of the population above 30 years of age deceasing every year	%
Pop >30	Fraction of the overall country population older than 30 years	%
YL	Average years of life loss per premature death between the ages of 30 and 70.	years

**Table A.6:** Correspondence table between SEAP and SHERPA sectors.

CoM Subsectors	Equivalent SHERPA MacroSector (MS)
Industry	Industry (MS 3)
Local Electricity Production	Industry (MS 1)
Local Heat Cold Production	Industry (MS 4)
Municipal Buildings Equipment Facilities	Buildings (Public, Residential and Tertiary, MS 2)
Others	Other: (MS 5, 6 and 9)
Public lighting	None
Residential Buildings	Buildings (Public, Residential and Tertiary, MS 2)
Tertiary Buildings	Buildings (Public, Residential and Tertiary)
Transport	Traffic

## Appendix A

This appendix provides a detailed description of data records extracted from the databases introduced in paragraph 2.2 and of the data merging and data cleaning procedures applied.

In merging the datasets, special care had to be taken in attributing the SEAP measures to a sector: as CoM and SHERPA have different numbers and definitions of sectors (9 CoM sectors vs 10 SHERPA sectors, corresponding to the SNAP CORINAIR standard (EMEP/EEA, 2016)) it has been necessary to map the CoM sectors into the SHERPA ones following the correspondences in Table A.6 In particular, the values of energy consumption in the CoM sectors obtained from the BEI database were summed up to obtain the energy consumption of each city in the SHERPA sectors. Finally four SHERPA sectors have been retained for the subsequent analysis.

Dataset merging led to the exclusion of a certain number of records because of incompleteness or inconsistency. In particular, the SCE and AQ datasets do not provide data for 12 of the 231 entries of the Cities dataset and the SCE dataset provides unreliable data for smaller cities as the resolution of the grid used in SHERPA is about 7 × 7 km^2^. Moreover, only SEAP measures for which both the estimated CO_2_ reduction and the energy consumed in the corresponding sector are reported were selected, in order to allow a proper estimate of the indicators described paragraph 2.3.

## Footnotes

1 The greater city is an approximation of the urban centres when this stretches far beyond the administrative city boundaries.

2 Each functional urban area is an economic unit characterised by densely inhabited “city core” and “commuting zone” whose labour market is highly integrated with the cores. For more details see i.e. http://www.oecd.org/cfe/regional-policy/functionalurbanareasbycountry.htm.

3 Whenever more suitable, values of AQB will be sometimes provided in percentage.

4 ANalysis Of VAriance (ANOVA) is a statistical methodology used to analyse the differences among group means. Here it is applied to investigate the actual difference of AQ and CC benefits provided by different sectorial subsets of ES measures.

5 ES measures belonging to the “Other” category were not analysed as an independent sector because of their heterogeneity.

6 In both cases differences between average values have been found statistically significant (p < 0.05) by means of ANOVA.

7 For the sector Industry, evidence for a linear trend was considered too weak, considering the much lower value of R^2^. For this sector it is likely that the heterogeneity of the actual industrial installations present in different cities results in the lack of a clear relation between CO_2_ emission reductions and AQ benefits.
